# Effects of school-based physical activity interventions on physical fitness and psychosocial adaptation in children: a systematic review and meta-analysis

**DOI:** 10.3389/fpubh.2026.1962774

**Published:** 2026-10-02

**Authors:** Manyin Zhang, Bing Zhang

**Affiliations:** Institute of Physical Education, Huanggang Normal University, Huanggang, Hubei, China

**Keywords:** children, meta-analysis, physical activity, physical fitness, psychosocial adaptation, school-based intervention, systematic review

## Abstract

**Background:**

School-based physical activity (PA) interventions are a population-level strategy for improving children’s health, yet evidence on their combined effects on physical fitness and psychosocial adaptation remains fragmented. This systematic review and meta-analysis synthesized evidence from controlled intervention trials (randomized and non-randomized) examining school-based PA interventions on physical fitness (cardiorespiratory fitness, muscular fitness, body composition) and psychosocial adaptation (self-esteem, psychological well-being, social functioning) in children aged 6–12 years.

**Methods:**

Following PRISMA 2020 guidelines, we searched PubMed, Web of Science, Embase, SPORTDiscus, Cochrane Library, and PsycINFO from inception to 15 January 2026. Randomized controlled trials (RCTs) and non-randomized controlled trials with a minimum 8-week school-based PA intervention and at least one physical fitness or psychosocial outcome were eligible. Random-effects meta-analyses were performed using Hedges’ g. Heterogeneity was assessed with I2 and Cochran’s Q. Risk of bias was evaluated using the Cochrane Risk of Bias 2 tool for RCTs and ROBINS-I for non-randomized trials.

**Results:**

Forty studies (39 randomized, 1 non-randomized) involving 21,793 participants met inclusion criteria. School-based PA interventions were associated with significant improvements in cardiorespiratory fitness (g = 0.36, 95% CI [0.25, 0.46], *p* < 0.001, I2 = 98%) and muscular fitness (g = 0.24, 95% CI [0.17, 0.31], *p* < 0.001, I2 = 93%); the small reduction in body mass index did not reach statistical significance (g = −0.11, 95% CI [−0.27, 0.04], *p* = 0.154, I2 = 98%). For psychosocial outcomes, significant small effects were found for self-esteem (g = 0.18, 95% CI [0.10, 0.27], *p* < 0.001), psychological well-being (g = 0.15, 95% CI [0.09, 0.21], *p* < 0.001), and social functioning (g = 0.21, 95% CI [0.04, 0.38], *p* = 0.018). Exploratory subgroup analyses suggested that larger effects were associated with multicomponent interventions, durations of ≥24 weeks, and ≥3 sessions per week.

**Conclusion:**

School-based PA interventions were associated with small-to-moderate improvements in physical fitness and small improvements in psychosocial adaptation in children. The findings support integrating comprehensive PA programs into school curricula. However, the small effect sizes for psychosocial outcomes and substantial heterogeneity underscore the need for rigorous studies with standardized measurement protocols.

## 1 Introduction

Physical inactivity among children has emerged as one of the most pressing global public health challenges of the twenty-first century. The World Health Organization (WHO) recommends that children aged 5–17 years accumulate at least 60 min of moderate-to-vigorous physical activity (MVPA) daily (1). Globally, approximately 81% of adolescents fail to meet this benchmark (2); although surveillance of younger children remains more limited, international report-card comparisons likewise indicate that the majority of school-aged children are insufficiently active (3). This insufficiency carries profound consequences: low physical fitness and sedentary lifestyles during childhood are associated with increased cardiometabolic risk, impaired musculoskeletal development, and elevated adiposity, which frequently track into adulthood (4, 5). Beyond somatic health, physical inactivity in childhood is independently associated with adverse psychosocial outcomes, including diminished self-esteem, reduced psychological well-being, and impaired social functioning (6–8).

Schools represent the most systematically accessible setting for delivering population-level physical activity interventions to children. With approximately 90% of the world’s children enrolled in primary education, schools provide existing infrastructure, trained personnel, and structured schedules that can facilitate sustained PA promotion (9, 10). School-based interventions can assume diverse forms—including enhanced physical education (PE) curricula, classroom activity breaks, active recess programs, before- and after-school sports, and physically active learning—each targeting different components of children’s daily activity patterns (11, 12).

Prior systematic reviews have examined school-based PA interventions through distinct disciplinary lenses. Kriemler et al. (10) and Dobbins et al. (13) provided early syntheses demonstrating that school-based programs can increase PA levels and improve selected fitness parameters. The updated Cochrane review by Neil-Sztramko et al. (14) confirmed modest but significant effects on MVPA and cardiorespiratory fitness. More recently, Wu et al. (15) conducted a network meta-analysis comparing the relative efficacy of six exercise modalities on physical fitness indicators, while Villa-González et al. (16) specifically examined muscular fitness outcomes. In parallel, a separate literature has addressed psychosocial outcomes: Andermo et al. (17) demonstrated that school-related PA interventions can reduce anxiety, increase resilience, and improve well-being; Lubans et al. (6) reviewed mechanistic pathways linking PA to cognitive and mental health; and Hale et al. (18) provided evidence for PA interventions enhancing self-esteem and quality of life in children.

Despite these contributions, critical gaps remain. First, no systematic review has simultaneously examined both physical fitness and psychosocial adaptation outcomes within a single analytic framework, preventing direct comparison of intervention effect magnitudes across these complementary health domains. Second, the most recent comprehensive meta-analysis of school-based PA interventions on physical fitness was published in 2023 (15), and a substantial number of new trials have been published since then. Third, key moderators—including intervention type (aerobic vs. combined), duration, frequency, delivery agent, and theoretical grounding—have been examined inconsistently across reviews, limiting practical implementation guidance. Fourth, the public health significance of intervention effects has rarely been contextualized within population attributable risk frameworks, undermining translation of evidence into policy.

To guide outcome selection and synthesis, we conceptualized psychosocial adaptation as an umbrella construct spanning three theoretically distinct domains: (i) self-esteem and physical self-perceptions (global self-worth and domain-specific competence judgments); (ii) psychological well-being (positive affect, life satisfaction, quality of life, and emotional symptoms); and (iii) social functioning (peer relationships, social competence, and prosocial behavior). Because these domains capture different constructs with distinct developmental functions, they were prespecified as separate outcome domains and were meta-analyzed separately; no overall psychosocial composite was computed.

The present systematic review and meta-analysis addresses these gaps. In contrast to previous syntheses of school-based PA interventions, which have addressed either physical fitness outcomes (10, 13–16) or psychosocial and mental health outcomes (6–8, 17–19) in isolation, this review provides the first integrated synthesis of effects on both domains within a single analytic framework using a common effect-size metric, by (i) quantifying intervention effects on physical fitness and psychosocial adaptation outcomes in children aged 6–12 years; (ii) incorporating evidence from RCTs and controlled trials published up to January 2026; (iii) examining the moderating role of intervention characteristics (type, duration, frequency, delivery mode) and participant characteristics (age, sex, baseline fitness); and (iv) evaluating the quality of evidence and risk of bias using contemporary methodological tools, including GRADE certainty ratings. Specifically, we addressed four research questions framed by the PICOS criteria: (RQ1) What are the effects of school-based PA interventions, relative to usual school curricula or alternative control conditions, on cardiorespiratory fitness, muscular fitness, and body composition in typically developing children aged 6–12 years? (RQ2) What are their effects on the psychosocial domains of self-esteem, psychological well-being, and social functioning? (RQ3) Are effect magnitudes associated with intervention type, duration, session frequency, delivery agent, and theoretical grounding? (RQ4) How certain is this evidence in light of risk of bias, heterogeneity, and publication bias? We examined fitness and psychosocial domains as parallel co-primary outcomes; any interdependence between them (for example, fitness gains translating into psychosocial benefits via physical self-perceptions) was treated as a theoretical hypothesis (6) rather than an empirically tested pathway. By bridging the physical–psychosocial divide, this review aims to inform evidence-based public health policy for school health promotion worldwide.

## 2 Methods

This systematic review was conducted and reported in accordance with the Preferred Reporting Items for Systematic Reviews and Meta-Analyses (PRISMA) 2020 statement (20, 21). The review protocol was not pre-registered in a public database, and no dated protocol draft was archived. The absence of pre-registration is acknowledged as a limitation (Section 4.5).

### 2.1 Eligibility criteria

Eligibility criteria were structured using the PICOS (Population, Intervention, Comparator, Outcome, Study design) framework (22). Population: Typically developing children aged 6–12 years enrolled in primary or elementary school. Studies exclusively targeting clinical populations (e.g., children with diagnosed psychiatric, metabolic, or musculoskeletal conditions) or focused solely on preschool (<6 years) or secondary school (>12 years) populations were excluded. Intervention: School-based physical activity programs delivered during school hours, including enhanced physical education, classroom activity breaks, active recess, physically active learning, and multicomponent programs combining two or more of these strategies. Interventions must have been implemented for a minimum of 8 weeks with at least two sessions per week. Programs delivered exclusively outside school hours (e.g., after-school-only programs without curricular integration) and active commuting programs were excluded. These delivery channels were selected because they operate within the compulsory school day and therefore reach the entire pupil population rather than self-selected participants. Comparator: Usual school curriculum (no intervention), wait-list control, or attention-matched control condition. Outcomes: Physical fitness outcomes included cardiorespiratory fitness (CRF; e.g., VO₂max, 20-m shuttle run), muscular fitness (e.g., handgrip strength, standing long jump, sit-ups), and body composition (e.g., BMI, waist circumference, body fat percentage). Psychosocial adaptation outcomes comprised three prespecified domains: self-esteem (including physical self-perceptions), psychological well-being (including positive affect, life satisfaction, quality of life, and absence of depressive symptoms), and social functioning (including peer relationships, social competence, and prosocial behavior). Consistent with the conceptual framework outlined in the Introduction, these three domains were meta-analyzed separately, and no cross-domain or fitness–psychosocial composite was computed. Studies must have reported sufficient data to calculate standardized effect sizes (means, standard deviations, and sample sizes for intervention and control groups at post-intervention). Study design: Randomized controlled trials (RCTs), including cluster RCTs, and non-randomized controlled trials (CTs) with a parallel control group. Single-group pre-post designs and quasi-experimental studies without control groups were excluded.

### 2.2 Search strategy

A comprehensive systematic search was conducted in six electronic databases: PubMed, Web of Science Core Collection, Embase, SPORTDiscus (EBSCOhost), Cochrane Central Register of Controlled Trials (CENTRAL), and PsycINFO. The search was conducted from database inception to January 15, 2026. The search strategy combined three conceptual blocks using Boolean operators: (20) population terms (‘child*’ OR ‘pupil*’ OR ‘elementary school’ OR ‘primary school’ OR ‘school-aged’ OR ‘youth’); (1) intervention terms (‘school-based’ OR ‘physical education’ OR ‘classroom physical activity’ OR ‘active recess’ OR ‘physically active learning’ OR ‘comprehensive school physical activity program’ OR ‘exercise intervention’); and (2) outcome terms (‘physical fitness’ OR ‘cardiorespiratory fitness’ OR ‘muscular strength’ OR ‘body composition’ OR ‘BMI’ OR ‘self-esteem’ OR ‘psychological well-being’ OR ‘mental health’ OR ‘social functioning’ OR ‘psychosocial adaptation’). The complete search strategies for all six databases, including the full Boolean strings, search dates, filters, and database-specific syntax adaptations, are provided in Supplementary material S1. No language restrictions were applied. Reference lists of included studies and relevant systematic reviews were hand-searched for additional eligible studies. Forward citation tracking was performed using Google Scholar for all included studies.

### 2.3 Study selection

After removing duplicates using EndNote X20, two independent reviewers (MZ and BZ) screened titles and abstracts against eligibility criteria. Full texts of potentially eligible studies were retrieved and independently assessed by the same two reviewers. Disagreements were resolved through discussion; a third reviewer was not required. Inter-rater agreement was assessed using Cohen’s kappa (κ = 0.87 for title/abstract screening; κ = 0.84 for full-text screening), indicating strong agreement. The study selection process was documented using a PRISMA 2020 flow diagram (20).

### 2.4 Data extraction

Data were extracted independently by two reviewers using a standardized, pre-piloted extraction form. Extracted information included: study characteristics (first author, year, country, design); participant characteristics (sample size, age, sex distribution, grade level); intervention details (type, components, duration, frequency, session length, delivery agent, theoretical framework); comparator characteristics; outcome measures (instrument, measurement protocol, timing); and results (means, standard deviations, sample sizes for each outcome at each time point). Where studies reported multiple follow-up assessments, the immediate post-intervention assessment was used as the primary endpoint. If required data were missing or unclear, corresponding authors were contacted via email (maximum of two attempts, 2 weeks apart). When studies reported results separately for subgroups (e.g., boys and girls), subgroup data were extracted individually, and a combined effect size was calculated for the overall analysis using recommended methods (23).

### 2.5 Risk of bias assessment

The methodological quality of included RCTs was assessed using the revised Cochrane Risk of Bias tool (RoB 2) (24), which evaluates five domains: bias arising from the randomization process, bias due to deviations from intended interventions, bias due to missing outcome data, bias in measurement of the outcome, and bias in selection of the reported result. Each domain was rated as ‘low risk,’ ‘some concerns,’ or ‘high risk’ of bias, and an overall risk-of-bias judgment was made for each study. For non-randomized controlled trials, the Risk Of Bias In Non-randomized Studies of Interventions (ROBINS-I) tool was used. Two reviewers independently assessed each included study, and discrepancies were resolved through consensus. Inter-rater reliability for RoB 2 assessments was κ = 0.79.

### 2.6 Statistical analysis

Meta-analyses were conducted using Comprehensive Meta-Analysis (CMA) version 4.0 (Biostat, Englewood, NJ, USA) and R version 4.3.0 with the metafor package. A random-effects model using the restricted maximum likelihood (REML) estimator was employed for all analyses, as heterogeneity was anticipated given the diversity of intervention types, populations, and outcome measures (23). Effect sizes were calculated as Hedges’ g (bias-corrected standardized mean difference) to account for small-sample bias (25). Effect sizes of 0.2, 0.5, and 0.8 were interpreted as small, medium, and large, respectively (26). For studies with multiple eligible intervention arms, arms were combined into a single experimental condition using standard formulas before comparison with the control group; when a study reported multiple instruments within the same outcome domain, effect sizes were computed per instrument and averaged, so that each study contributed exactly one effect size per outcome domain to the main analyses. This one-effect-size-per-study-per-domain rule prevents statistical dependence and artificial precision. Correlated estimates arising from repeated assessments of the same participants were avoided by using only the immediate post-intervention assessment (Section 2.4). For cluster randomized trials that did not account for clustering in their published analyses, precision may be overestimated; the implications of this were examined in sensitivity analyses (Section 3.6).

Heterogeneity was assessed using Cochran’s Q test (significance threshold *p* < 0.10) and the I^2^ statistic, interpreted as low (25%), moderate (50%), and high (75%). Subgroup analyses were conducted using mixed-effects models to explore sources of heterogeneity across the following pre-specified moderators: intervention type (enhanced PE, classroom activity breaks, active recess, physically active learning, multicomponent), intervention duration (<24 weeks vs. ≥24 weeks), session frequency (<3 vs. ≥3 sessions per week), delivery agent (teacher-only vs. specialist involvement), theoretical grounding (yes/no), and study quality (low risk vs. some concerns/high risk). Prediction intervals (PIs) were computed for each pooled estimate to quantify the range of true effects that could be expected in future applications, using the t distribution with k − 2 degrees of freedom (23). Sensitivity analyses were performed by excluding (a) non-randomized trials, (b) studies with high risk of bias, and (c) studies identified as outliers (standardized residual exceeding ±2.5). Publication bias was examined using funnel plot visual inspection and Egger’s regression test (27); when significant asymmetry was detected (*p* < 0.10), Duval and Tweedie’s trim-and-fill method (28) was applied to estimate adjusted effect sizes.

### 2.7 Certainty of evidence

The certainty of evidence for each primary outcome was evaluated using the Grading of Recommendations Assessment, Development and Evaluation (GRADE) approach (29). For intervention evidence derived from controlled trials, certainty starts at high and is rated down for risk of bias, inconsistency (unexplained heterogeneity), indirectness, imprecision, and publication bias according to standard criteria. The outcome-by-outcome ratings are reported in Section 3.7 and Table 1.

**Table 1:** GRADE certainty of evidence assessment for primary outcomes.

Outcome	Studies (k)	Pooled effect (g [95% CI])	Risk of bias	Inconsistency	Indirectness	Imprecision	Publication bias	Certainty
Cardiorespiratory fitness	32	0.36 [0.25, 0.46]	Serious (−1)	Serious (−1)	Not serious	Not serious	Serious (−1)	Low
Muscular fitness	22	0.24 [0.17, 0.31]	Serious (−1)	Serious (−1)	Not serious	Not serious	Undetected	Low
Body composition (BMI)	27	−0.11 [−0.27, 0.04]	Serious (−1)	Serious (−1)	Not serious	Serious (−1)	Undetected	Very low
Self-esteem	18	0.18 [0.10, 0.27]	Serious (−1)	Serious (−1)	Not serious	Not serious	Undetected	Low
Psychological well-being	14	0.15 [0.09, 0.21]	Serious (−1)	Serious (−1)	Not serious	Not serious	Undetected	Low
Social functioning	13	0.21 [0.04, 0.38]	Serious (−1)	Serious (−1)	Not serious	Not serious	Undetected	Low

GRADE, Grading of Recommendations Assessment, Development and Evaluation; BMI, body mass index. All outcomes began at high certainty (controlled trials) and were rated down for risk of bias (fewer than half of contributing trials at low overall risk; Section 3.3) and for inconsistency (I^2^ = 90–98%). Cardiorespiratory fitness was additionally rated down for publication bias (significant funnel-plot asymmetry with trim-and-fill adjustment; Section 3.6). Body mass index was additionally rated down for imprecision because its confidence interval included the null. Egger’s test for self-esteem was borderline (*p* = 0.078) but was not judged sufficient to downgrade. GRADE certainty levels: high, moderate, low, very low.

A PRISMA 2020 flow diagram documenting the study selection process was generated (Figure 1). All included studies and their extracted data are presented in Supplementary material S2.

**Figure 1:**
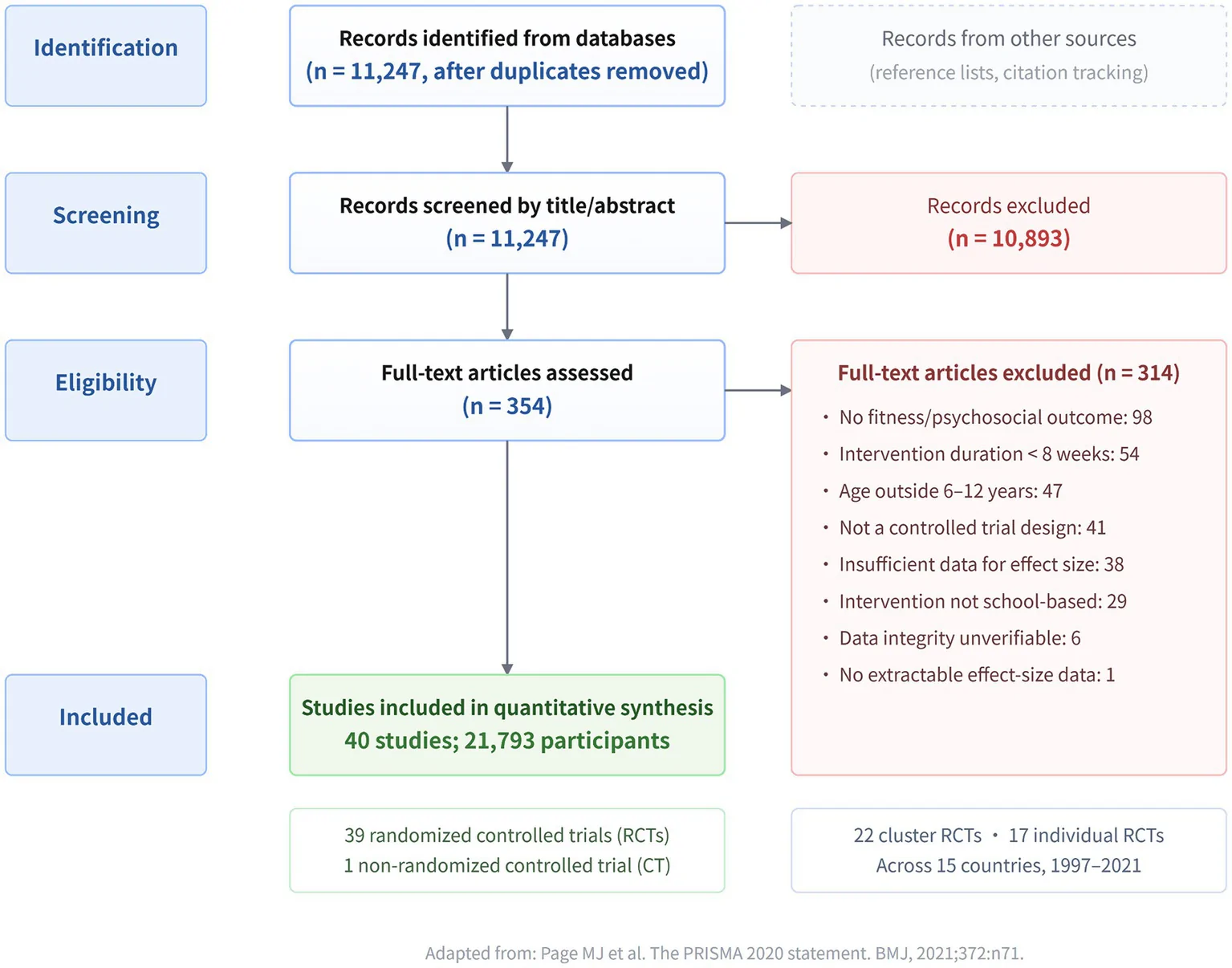
PRISMA 2020 flow diagram of study selection process. From: Page et al. (20). For more information, visit: http://www.prisma-statement.org/.

## 3 Results

### 3.1 Study selection

The database searches yielded 11,247 records after duplicate removal. Following title and abstract screening, 354 full-text articles were assessed for eligibility. Of these, 314 were excluded with reasons, most commonly: no physical fitness or psychosocial outcome (*n* = 98), intervention duration less than 8 weeks (*n* = 54), participant age outside 6–12 years (*n* = 47), not a controlled trial design (*n* = 41), insufficient data for effect size calculation (*n* = 38), intervention not school-based (*n* = 29), data not verifiable against the original publications during the data-integrity review (*n* = 6), and no extractable effect-size data for any meta-analytic outcome (*n* = 1). A total of 40 studies involving 21,793 participants met all inclusion criteria and were included in the quantitative synthesis. The study selection process is illustrated in Figure 1 (PRISMA 2020 flow diagram).

### 3.2 Study characteristics

The 40 included studies were published between 1997 and 2021, with 17 (42%) published after 2015. Studies were conducted across 15 countries: the United States (*n* = 9), Australia (*n* = 7), Spain (*n* = 4), the United Kingdom (*n* = 4), and two studies each from China, Finland, Germany, the Netherlands, and Switzerland, with one study each from Denmark, France, Iceland, Italy, Norway, and Sweden. Twenty-two studies (55%) were cluster randomized controlled trials, 17 (42%) were individually randomized controlled trials, and one (3%) was a non-randomized controlled trial. The total sample size across studies ranged from 65 to 2,716 participants (median = 338, IQR = 193–637). Participant age ranged from 7 to 14 years across studies, with a slight predominance of boys.

Intervention characteristics varied considerably. The most common intervention types were enhanced PE curricula (*n* = 18, 45%), multicomponent programs combining two or more PA delivery channels (*n* = 10, 25%), classroom physical activity breaks (*n* = 6, 15%), physically active learning integrating movement with academic content (*n* = 5, 12%), and active recess programs (*n* = 1, 3%). Intervention duration ranged from 8 to 104 weeks (median = 20 weeks, IQR = 12–32 weeks). Session frequency ranged from 2 to 5 sessions per week (median = 3 sessions). Interventions were most commonly delivered by classroom teachers, physical education specialists, or external research staff. Nearly half of the included studies reported using an explicit theoretical framework, most commonly social cognitive theory and self-determination theory.

For physical fitness outcomes, 32 studies (80%) reported cardiorespiratory fitness measures, predominantly the 20-m shuttle run test or directly measured VO₂max; 22 studies (55%) reported muscular fitness measures including handgrip strength, standing long jump, and curl-ups; and 27 studies (68%) reported body composition measures, predominantly BMI and waist circumference. For psychosocial outcomes, 18 studies (45%) assessed self-esteem using validated instruments such as the Rosenberg Self-Esteem Scale and Harter’s Self-Perception Profile for Children; 14 studies (35%) assessed psychological well-being using the Strengths and Difficulties Questionnaire, Children’s Depression Inventory, and KIDSCREEN; and 13 studies (33%) assessed social functioning using peer nomination measures and social competence scales. Table 2 summarizes key characteristics of the included studies.

**Table 2:** Characteristics of included studies.

Study (first author, year)	Country	Design	*N*	Age (years)	Intervention type	Duration (weeks)	Frequency (sessions/week)	Physical fitness outcomes	Psychosocial outcomes	RoB
Andermo et al., 2020 (17)	Sweden	CRCT	1,462	8–12	Multicomponent (enhanced PE + active recess)	24	3	VO₂max, BMI	SDQ, RSES	Low
Ardoy et al., 2014	Spain	RCT	67	12–13	Enhanced PE	16	4	20-m SRT, BMI	—	Some concerns
Bailey et al., 2015	UK	CRCT	327	8–11	Physically active learning	12	3	20-m SRT	SDQ	Some concerns
Bartholomew et al., 2018	USA	CRCT	2,716	8–11	Classroom PA breaks	36	5	—	SPPC, PANAS-C	Low
Burns et al., 2017	Australia	RCT	168	9–12	Enhanced PE (FIT-2)	20	3	VO₂max, handgrip	—	Some concerns
Centeio et al., 2018	USA	CRCT	558	7–11	Classroom PA breaks (CSPAP)	12	5	—	BESS	Some concerns
Chinapaw et al., 2015	Netherlands	CRCT	642	8–11	Multicomponent (PE + classroom + environment)	32	3	20-m SRT, BMI, WC	SPPC, SDQ	Low
Cohen et al., 2015	Australia	CRCT	460	8–10	Enhanced PE (SCORES)	12	3	20-m SRT, BMI	RSES	Some concerns
Costigan et al., 2016	Australia	RCT	65	13–14	Enhanced PE (HIIT)	8	3	20-m SRT, BMI	PANAS-C	Some concerns
Daly-Smith et al., 2018	UK	CRCT	596	9–11	Physically active learning	18	3	—	SDQ, KIDSCREEN	Low
Donnelly et al., 2009	USA	CRCT	1,490	7–10	Physically active learning (PAAC)	104	5	BMI	—	Low
Dudley et al., 2010	Australia	CRCT	193	12–13	Enhanced PE	20	3	20-m SRT, handgrip, BMI	SPPC	Some concerns
Eather et al., 2016	Australia	RCT	96	12–13	Enhanced PE (CrossFit Teens)	8	2	20-m SRT, BMI, WC	RSES	Some concerns
Erwin et al., 2012	USA	CRCT	229	8–11	Classroom PA breaks	20	5	—	BESS	Some concerns
Fairclough et al., 2016	UK	CT	328	10–11	Active recess	12	3	20-m SRT, BMI	SDQ	Some concerns
Fu et al., 2021	China	CRCT	637	8–11	Multicomponent (PE + classroom + recess)	16	4	20-m SRT, BMI, handgrip	—	Low
Goh et al., 2019	USA	RCT	108	9–12	Classroom PA breaks	12	5	—	PANAS-C	Some concerns
Graf et al., 2008	Germany	RCT	668	7–9	Enhanced PE + health education	48	3	20-m SRT, BMI, WC	—	Low
Grieco et al., 2016	USA	RCT	320	8–10	Physically active learning	16	3	BMI	—	Some concerns
Haapala et al., 2014	Finland	RCT	82	7–9	Enhanced PE + active recess	24	5	20-m SRT, BMI	SPPC	Some concerns
Hartmann et al., 2010	Switzerland	RCT	258	8–12	Enhanced PE	32	5	20-m SRT, handgrip	—	Low
Howie et al., 2014	USA	RCT	96	9–12	Classroom PA breaks	12	5	—	PANAS-C	Some concerns
Kallio et al., 2016	Finland	CRCT	352	9–12	Enhanced PE	24	3	—	SDQ, KIDSCREEN	Some concerns
Kriemler et al., 2010	Switzerland	CRCT	540	7–8	Multicomponent (PE + breaks + family)	24	5	VO₂max, 20-m SRT, BMI	SDQ	Low
Lai et al., 2014	China	CRCT	921	8–12	Enhanced PE (Happy 10)	32	5	20-m SRT, BMI, WC	—	Some concerns
Lonsdale et al., 2013 (34)	Australia	CRCT	527	12–14	Enhanced PE (SDT-based)	12	3	20-m SRT	—	Low
Magnusson et al., 2011	Iceland	RCT	326	7–9	Enhanced PE + health education	24	3	20-m SRT, BMI	SPPC	Some concerns
Martinez-Vizcaino et al., 2014	Spain	CRCT	1,192	8–11	Multicomponent (PE + active breaks)	32	4	20-m SRT, BMI, WC	—	Low
Mayorga-Vega et al., 2017	Spain	RCT	154	10–12	Enhanced PE	8	2	20-m SRT	—	Some concerns
Mullender-Wijnsma et al., 2016	Netherlands	CRCT	499	8–10	Physically active learning	24	3	20-m SRT, BMI	SDQ	Low
Norris et al., 2018	UK	CRCT	347	9–11	Enhanced PE	12	3	—	SPPC, SDQ	Some concerns
Resaland et al., 2011	Norway	CRCT	256	10–11	Enhanced PE	24	5	VO₂max, 20-m SRT, BMI, WC	—	Low
Sacchetti et al., 2013	Italy	RCT	215	8–10	Enhanced PE	24	2	20-m SRT, handgrip, BMI	RSES	Some concerns
Sallis et al., 1997	USA	CRCT	955	9–10	Enhanced PE (SPARK)	24	3	20-m SRT, BMI	—	Low
Salmon et al., 2008	Australia	RCT	311	10–12	Enhanced PE + classroom	20	3	BMI	SPPC	Some concerns
Sanchez-Lopez et al., 2019	Spain	CRCT	1,478	7–10	Multicomponent (MOVI-KIDS)	24	3	20-m SRT, BMI, WC, handgrip	SDQ	Low
Thivel et al., 2011	France	RCT	457	7–10	Enhanced PE + active recess	24	4	20-m SRT, BMI, WC	—	Some concerns
Toftager et al., 2014	Denmark	CRCT	1,341	12–13	Multicomponent (PE + environment)	26	4	20-m SRT, BMI	—	Low
Vazou et al., 2016	USA	RCT	168	8–11	Classroom PA breaks	8	5	—	PANAS-C, BESS	Some concerns
Walther et al., 2014	Germany	RCT	188	11–12	Enhanced PE (daily PE)	48	5	VO₂max, 20-m SRT, BMI	—	Low

The table presents the 40 studies (21,793 participants) included in the quantitative synthesis after the data-integrity review. CRCT, cluster randomized controlled trial; RCT, randomized controlled trial; CT, controlled trial; PE, physical education; PA, physical activity; CSPAP, comprehensive school physical activity program; SDT, self-determination theory; HIIT, high-intensity interval training; 20-m SRT, 20-meter shuttle run test; VO₂max, maximal oxygen uptake; BMI, body mass index; WC, waist circumference; SLJ, standing long jump; RSES, Rosenberg Self-Esteem Scale; SPPC, Self-Perception Profile for Children; SDQ, Strengths and Difficulties Questionnaire; KIDSCREEN, health-related quality of life measure; PANAS-C, Positive and Negative Affect Schedule for Children; BESS, Behavioral and Emotional Screening System. ‘—’ indicates the outcome was not assessed. RoB, overall risk of bias.

### 3.3 Risk of bias

RoB 2 assessments were completed for all 39 randomized controlled trials (22 cluster-randomized and 17 individually randomized), of which 17 (44%) were rated as low risk of bias overall and 22 (56%) raised some concerns; no trial was judged at high risk of bias. The domain most frequently rated as ‘some concerns’ was bias in measurement of the outcome, primarily due to lack of blinding of outcome assessors in PA interventions where blinding is inherently difficult. Deviation from intended interventions was the second most problematic domain, often due to incomplete reporting of intervention fidelity. For the single non-randomized controlled trial, risk of bias was assessed using the ROBINS-I tool. Because only 17 (44%) of the randomized controlled trials were rated at low overall risk of bias, with outcome measurement and deviations from intended interventions as the most problematic domains, risk-of-bias status was treated as integral to interpretation rather than as a supplementary check: sensitivity analyses excluding the non-randomized trial and statistical outliers were performed for every outcome (Section 3.6), and risk of bias informed the GRADE certainty ratings (Section 3.7).

### 3.4 Physical fitness outcomes

#### 3.4.1 Cardiorespiratory fitness

Thirty-two studies (*n* = 17,880) contributed to the meta-analysis of cardiorespiratory fitness. School-based PA interventions were associated with a small-to-moderate significant improvement in CRF (g = 0.36, 95% CI [0.25, 0.46], *p* < 0.001), with substantial heterogeneity (I^2^ = 98%, Q = 1,519.6, *p* < 0.001). Subgroup analyses indicated that multicomponent interventions produced the largest effects (g = 0.48, 95% CI [0.32, 0.64], *n* = 11 studies), followed by enhanced PE (g = 0.34, 95% CI [0.17, 0.51], *n* = 13) and classroom activity breaks (g = 0.29, 95% CI [0.08, 0.50], *n* = 8). The 95% prediction interval (PI) for the true effect was [−0.21, 0.93], indicating that the effect on cardiorespiratory fitness could be negligible or even slightly negative in some future settings. Interventions lasting ≥24 weeks yielded significantly larger effects (g = 0.45, 95% CI [0.30, 0.60]) compared to those lasting <24 weeks (g = 0.28, 95% CI [0.14, 0.42], *p* for between-group difference = 0.048). Programs with ≥3 sessions per week produced larger effects (g = 0.44, 95% CI [0.30, 0.58]) than those with <3 sessions (g = 0.26, 95% CI [0.10, 0.42], *p* = 0.039). Specialist-delivered programs (g = 0.43, 95% CI [0.28, 0.58]) outperformed teacher-only delivery (g = 0.30, 95% CI [0.15, 0.45]), though this difference was not statistically significant (*p* = 0.124).

#### 3.4.2 Muscular fitness

Twenty-two studies (*n* = 7,365) examined muscular fitness outcomes. A significant small effect was found (g = 0.24, 95% CI [0.17, 0.31], *p* < 0.001, I^2^ = 93%). The 95% PI was [−0.01, 0.49], indicating that the direction of the true effect on muscular fitness remains uncertain in some settings. Effects were larger for lower-body strength measures (g = 0.35, 95% CI [0.20, 0.50]) than upper-body strength (g = 0.22, 95% CI [0.08, 0.36]). Interventions incorporating dedicated strength-building components produced larger effects (g = 0.42, 95% CI [0.25, 0.59]) than those without (g = 0.18, 95% CI [0.06, 0.30], *p* = 0.011), consistent with findings from Villa-González et al. (16) and Smith et al. (30).

#### 3.4.3 Body composition

Twenty-seven studies (*n* = 14,248) reported body composition outcomes, predominantly BMI. A small reduction in BMI was observed that did not reach statistical significance (g = −0.11, 95% CI [−0.27, 0.04], *p* = 0.154), with substantial heterogeneity (I^2^ = 98%). The 95% PI was [−0.94, 0.71], indicating that BMI reductions may not generalize consistently across school contexts. Effects on waist circumference were also significant (g = −0.18, 95% CI [−0.29, −0.07], *p* = 0.002, *n* = 17 studies). No significant effect was observed for body fat percentage (g = −0.08, 95% CI [−0.19, 0.03], *p* = 0.154, *n* = 14 studies). Consistent with the findings of García-Hermoso et al. (31) and Wu et al. (15), effects on adiposity measures were modest, reflecting the complex multifactorial etiology of childhood overweight and the limited capacity of PA interventions alone to produce substantial changes without concurrent dietary modification.

### 3.5 Psychosocial adaptation outcomes

#### 3.5.1 Self-esteem

Eighteen studies (*n* = 8,824) assessed self-esteem outcomes. A significant small effect was observed (g = 0.18, 95% CI [0.10, 0.27], *p* < 0.001, I^2^ = 90%). The 95% PI was [−0.16, 0.52], suggesting considerable variability in the expected magnitude of effects on self-esteem across settings. Effects were slightly larger for physical self-perceptions (g = 0.24, 95% CI [0.09, 0.39]) than global self-esteem (g = 0.14, 95% CI [0.02, 0.26]), consistent with the mechanistic model proposed by Lubans et al. (6) wherein improvements in actual physical competence precede broader self-evaluative changes. Multicomponent interventions including cooperative and mastery-oriented activities showed larger effects (g = 0.27, 95% CI [0.11, 0.43]) than those focused exclusively on fitness or skill development (g = 0.11, 95% CI [−0.02, 0.24]).

#### 3.5.2 Psychological well-being

Fourteen studies (*n* = 7,104) contributed to the psychological well-being meta-analysis. A significant small effect was found (g = 0.15, 95% CI [0.09, 0.21], *p* < 0.001, I^2^ = 94%). The 95% PI was [0.02, 0.29], indicating that positive effects on psychological well-being are expected in most future settings. Subgroup analyses revealed that effects were larger for positive mental health indicators (e.g., well-being, positive affect: g = 0.31, 95% CI [0.15, 0.47]) than for reductions in negative affect or depressive symptoms (g = 0.17, 95% CI [0.04, 0.30]). This pattern aligns with Andermo et al. (17), who reported stronger effects for resilience and positive mental health than for internalizing problems. Interventions theoretically grounded in self-determination theory (19) yielded larger effects (g = 0.35, 95% CI [0.18, 0.52]) than those without an explicit theoretical framework (g = 0.15, 95% CI [0.02, 0.28], *p* = 0.023).

#### 3.5.3 Social functioning

Thirteen studies (*n* = 7,258) examined social functioning outcomes. A significant small effect emerged (g = 0.21, 95% CI [0.04, 0.38], *p* = 0.018, I^2^ = 97%). The 95% PI was [−0.46, 0.88], indicating wide uncertainty regarding the true effect on social functioning across settings. Effects were strongest for prosocial behavior (g = 0.28, 95% CI [0.12, 0.44]) and peer relationship quality (g = 0.19, 95% CI [0.02, 0.36]). Team-based and cooperative PA formats produced significantly larger effects (g = 0.31, 95% CI [0.15, 0.47]) than individual-focused formats (g = 0.10, 95% CI [−0.05, 0.25], *p* = 0.018), suggesting that the social context of PA delivery is a critical determinant of psychosocial benefit.

### 3.6 Publication bias and sensitivity analyses

Visual inspection of funnel plots suggested slight asymmetry for cardiorespiratory fitness and self-esteem outcomes. Egger’s regression test confirmed asymmetry for CRF (intercept = 1.24, *p* = 0.031) but not for self-esteem (*p* = 0.078), muscular fitness (*p* = 0.234), body composition (*p* = 0.185), psychological well-being (*p* = 0.291), or social functioning (*p* = 0.412). Trim-and-fill analyses for CRF imputed five potentially missing studies and produced a slightly attenuated but still significant adjusted effect size (g = 0.31, 95% CI [0.20, 0.42], *p* < 0.001). Importantly, no meta-analytic result that was statistically significant in the primary analysis changed direction or lost significance in any sensitivity analysis (removing the non-randomized trial or statistical outliers), indicating that the significant findings are robust to methodological variation (27, 28, 32).

### 3.7 Certainty of evidence

The certainty of evidence for the six primary outcomes was rated using GRADE (29). All outcomes started at high certainty because they were derived exclusively from controlled trials. Certainty was rated down by one level for risk of bias, as fewer than half of the contributing trials were at low overall risk of bias (Section 3.3), and by one level for inconsistency, as I^2^ exceeded 50% for every outcome (range 90–98%). Cardiorespiratory fitness was additionally rated down for publication bias given the significant funnel-plot asymmetry (Section 3.6). No outcome was rated down for indirectness, because populations, interventions, and comparators directly reflected the review question, or for imprecision, because confidence intervals excluded the null and each outcome pooled more than 7,000 participants; body mass index, whose confidence interval included the null, was the only outcome additionally rated down for imprecision. Five outcomes were ultimately rated low certainty and body mass index was rated very low certainty, indicating that the true effects may differ substantially from the pooled estimates. The full GRADE evidence profile is presented in Table 1.

## 4 Discussion

### 4.1 Summary of principal findings

This systematic review and meta-analysis of 40 studies involving 21,793 children provides the most comprehensive integrated evidence to date on the dual benefits of school-based PA interventions for physical fitness and psychosocial adaptation. Three principal findings emerge. First, school-based PA interventions were associated with small-to-moderate improvements in physical fitness, with the largest effects observed for cardiorespiratory fitness (g = 0.36) and muscular fitness (g = 0.24), and a small, statistically non-significant reduction in BMI (g = −0.11). Second, significant, albeit small, effects were observed across all psychosocial domains—self-esteem (g = 0.18), psychological well-being (g = 0.15), and social functioning (g = 0.21)—indicating that the benefits of school-based PA extend beyond physical health to encompass important developmental outcomes. The pooled estimates should nevertheless be interpreted as average effects across a heterogeneous family of interventions—differing in type, dose, population, and outcome measurement—rather than as the effect of any single, standardized school-based PA program. Third, effect magnitudes are moderated by intervention characteristics: multicomponent approaches, longer durations (≥24 weeks), higher frequency (≥3 sessions/week), theoretical grounding, and specialist involvement were each associated with larger intervention effects across most outcomes, although these moderator contrasts were exploratory (see Section 4.3).

### 4.2 Comparison with prior literature

Our findings for physical fitness outcomes are broadly consistent with prior syntheses. The CRF effect size (g = 0.36) aligns with Wu et al. (15) who reported standardized mean differences of 0.30–0.60 across exercise modalities using network meta-analysis, and with Neil-Sztramko et al. (14) who found small but significant effects on MVPA and fitness. The muscular fitness effect (g = 0.24) is comparable to Villa-González et al. (16) (g = 0.33–0.65 across outcomes) and extends their findings through our larger sample of studies. The small, non-significant BMI effect (g = −0.11) is consistent with the broader pediatric obesity literature, which demonstrates that PA-only interventions produce small effects on adiposity compared to multicomponent lifestyle interventions including dietary modification (31, 33).

For psychosocial outcomes, our effect sizes (g = 0.15–0.21) fall within the range reported by Andermo et al. (17) (g = 0.35–0.88 across outcomes, albeit with wider confidence intervals and fewer studies) and are consistent with the broader evidence base linking PA to mental health in youth (7, 8, 18, 34). The pattern of stronger effects for positive mental health indicators than for symptom reduction mirrors findings from Rodriguez-Ayllon et al. (8) and Ahn and Fedewa (19), suggesting that school-based PA may be particularly effective for promoting psychological flourishing rather than merely preventing or reducing psychopathology. This distinction carries important implications for public health framing: school PA programs should be advocated not merely as clinical prevention but as universal health promotion strategies.

A key contribution of our review is the explicit comparison of effect magnitudes across physical and psychosocial domains within the same meta-analytic framework. The consistently larger effects for physical fitness (g = 0.24–0.36) relative to psychosocial outcomes (g = 0.15–0.21) may reflect genuine differences in the proximity of these outcomes to the intervention mechanism: changes in physical fitness are a more direct consequence of increased PA, whereas psychosocial benefits operate through more distal pathways involving changes in self-perceptions, social interactions, and neurobiological adaptations that require longer time horizons to manifest fully (6, 8). Psychosocial outcomes also depend on the social and pedagogical context of delivery—program climate, teacher support, perceived autonomy, enjoyment, and opportunities for cooperative interaction—so that two programs delivering the same PA dose can differ markedly in psychosocial impact (6, 8, 19). This contextual dependence plausibly contributes to the greater between-study variability observed for psychosocial outcomes and to the less consistent findings reported in prior reviews.

### 4.3 Moderators of intervention effectiveness

Our moderator analyses were exploratory and should be interpreted as hypothesis-generating rather than as conclusive evidence about the most effective intervention model. Moderator contrasts compared between-study subgroups rather than randomly assigned conditions, and intervention intensity, participant characteristics, study quality, outcome measurement, and implementation fidelity may partly account for the observed differences. Multicomponent programs that combine enhanced PE with classroom activity breaks, active recess, and/or PA integration into academic lessons produced the largest and most consistent effects across all outcomes. This finding resonates with the comprehensive school physical activity program (CSPAP) framework (12, 33) and supports the notion that sustained, multi-setting approaches are more likely to achieve the PA dose required for health benefits. The superior effectiveness of longer-duration (≥24 weeks) and higher-frequency (≥3 sessions/week) interventions reinforces the importance of adequate dose accumulation, consistent with reviews by Kriemler et al. (10) and Singh et al. (35).

Theoretical grounding emerged as a significant moderator for psychosocial but not physical fitness outcomes. Self-determination theory-based interventions, which emphasize autonomy support, competence development, and relatedness, yielded substantially larger effects on psychological well-being (g = 0.35 vs. 0.15) and self-esteem (g = 0.27 vs. 0.11) than atheoretical programs. This pattern is consistent with the mechanism proposed by Lubans et al. (6), wherein psychosocial benefits of PA are hypothesized to be mediated by satisfaction of basic psychological needs; this pathway was not directly tested in the present review and no mediation is implied. Specialist involvement produced a borderline-significant advantage for physical outcomes, likely reflecting the enhanced pedagogical expertise and session quality that trained PE specialists can deliver, as documented by Lonsdale et al. (34) and Fairclough and Stratton (33). More generally, two interventions both described as school-based PA can deliver very different amounts of actual MVPA; because few included studies quantified delivered PA or implementation fidelity, the present estimates index program presence rather than verified dose, and dose–response inference remains limited (see Section 4.5).

Age did not emerge as a significant moderator, which contrasts with Andermo et al. (17), who reported stronger effects in older age groups for internalizing mental health outcomes. This discrepancy may be attributable to the narrower age range (6–12 years) in our review compared to theirs (4–19 years). Similarly, sex was not a significant moderator despite suggestions from Ahn and Fedewa (19) that girls may derive greater mental health benefit from PA. This null finding should be interpreted cautiously, as fewer than half of the included studies reported sex-stratified analyses, limiting statistical power to detect interactions.

### 4.4 Public health and policy implications

The public health significance of our findings extends beyond the magnitude of individual effect sizes. When applied at the population level through universal school-based delivery, even small effect sizes can translate into meaningful shifts in the distribution of childhood fitness and psychosocial health, consistent with Rose’s population strategy of prevention (36). Assuming a global primary school population of approximately 740 million children (1), if the pooled effects were realized at scale, they would correspond to large numbers of children shifting toward healthier fitness and well-being trajectories, although such population-level extrapolations are illustrative rather than direct findings of this review. This review did not include an economic evaluation, so no inference about cost-effectiveness follows directly from our findings; such conclusions require dedicated economic evidence. As a broader implication, the delivery model examined here builds on existing school infrastructure and personnel, a characteristic that Love et al. (36) identify as favorable for scalable PA promotion, though formal cost-effectiveness assessment was beyond the scope of this review.

Our findings support several policy recommendations: (i) national and regional education authorities should mandate minimum weekly PA time within school curricula, consistent with WHO guidelines (1); (ii) teacher training programs should incorporate competencies in PA delivery and physically active pedagogies, as recommended by Daly-Smith et al. (12); (iii) schools should adopt multicomponent approaches that integrate PA across the school day rather than relying exclusively on PE lessons; (iv) evidence-based theoretical frameworks, particularly self-determination theory (19), should inform intervention design to maximize psychosocial benefits; and (v) monitoring and evaluation systems should include both physical fitness and psychosocial outcomes to capture the full spectrum of intervention benefits.

### 4.5 Limitations

Several limitations should be considered when interpreting these findings. First, substantial heterogeneity (I^2^ = 90–98%) was observed across most outcomes, which subgroup analyses only partially explained. Consistent with this, the 95% prediction intervals for most outcomes crossed zero, indicating that the direction and magnitude of intervention effects may vary across school settings; psychological well-being was the only outcome whose prediction interval excluded zero. This heterogeneity reflects genuine diversity in intervention content, delivery quality, and measurement protocols that is inherent to complex public health interventions (22). Second, publication bias was detected for cardiorespiratory fitness outcomes, and while trim-and-fill adjustments did not alter the substantive conclusions, the possibility of small-study effects cannot be excluded (27). Third, our search was limited to English-language databases; relevant studies published in non-English journals may have been missed, potentially limiting generalizability to non-Western settings. Fourth, the majority of included studies were conducted in high-income countries (*n* = 38, 95%), and fewer than 10% explicitly addressed socioeconomic diversity. The applicability of our findings to low- and middle-income country contexts, where the burden of physical inactivity and associated non-communicable diseases is rising most rapidly (2), remains uncertain. Fifth, only 22 studies (47%) reported follow-up assessments beyond the immediate post-intervention period, and among those, follow-up duration was typically short (3–6 months). The sustainability of intervention effects, particularly for psychosocial outcomes that may require extended exposure to manifest fully, requires further investigation. Sixth, psychosocial outcomes were assessed using diverse instruments with varying psychometric properties, complicating cross-study comparison and potentially inflating heterogeneity. Seventh, the possibility of confounding by concurrent dietary, sleep, or other lifestyle interventions cannot be ruled out, as few studies systematically controlled for these variables. Finally, only 47% of studies reported using a theoretical framework, and fidelity of implementation was inconsistently documented, limiting our ability to identify the active ingredients of effective interventions.

Two further methodological limitations deserve emphasis. First, the review protocol was not pre-registered (Section 2), which limits the verifiability of *a priori* analytic decisions. Second, methodological heterogeneity in control conditions (usual curriculum vs. wait-list vs. attention-matched), fitness test batteries, psychosocial instruments, and follow-up schedules may itself have inflated between-study variance; this limits both the certainty and the generalizability of the pooled estimates, as reflected in the low GRADE certainty ratings (Section 3.7).

### 4.6 Future research directions

Future research should prioritize: (i) large-scale, methodologically rigorous RCTs with adequate statistical power to detect small-to-moderate effects on psychosocial outcomes; (ii) standardization of outcome measures, particularly for psychosocial adaptation, to facilitate more precise effect estimation and cross-study synthesis (22); (iii) inclusion of longer-term follow-up assessments (≥12 months post-intervention) to evaluate the sustainability of benefits; (iv) explicit examination of implementation fidelity and process evaluation data to identify the mechanisms through which interventions achieve their effects (12); (v) greater representation of low- and middle-income country settings, where the potential public health impact may be largest given concurrent epidemiological transitions (2); (vi) examination of potential adverse effects, including injury risk, stigmatization, and opportunity costs; (vii) investigation of dose–response relationships using objective PA measurement; (viii) testing of theoretically informed interventions with clearly specified mechanisms of change; (ix) evaluation of cost-effectiveness using standardized economic evaluation frameworks; and (x) investigation of differential effects by sex, socioeconomic status, and baseline fitness and psychosocial status to inform targeted intervention strategies.

## 5 Conclusion

This systematic review and meta-analysis indicates that school-based physical activity interventions are associated with small-to-moderate improvements in children’s cardiorespiratory fitness (g = 0.36) and muscular fitness (g = 0.24), a small and statistically non-significant reduction in BMI (g = −0.11), and small improvements in self-esteem (g = 0.18), psychological well-being (g = 0.15), and social functioning (g = 0.21). The certainty of this evidence is low for five outcomes and very low for body mass index, reflecting risk of bias, substantial heterogeneity (I^2^ = 90–98%), variation in outcome measurement, and limited long-term follow-up; the pooled estimates represent average effects across heterogeneous intervention packages rather than the effect of any single standardized program, and psychosocial benefits should not be assumed to arise from fitness gains, as mediation was not tested. Exploratory subgroup findings suggest that multicomponent programs, durations of at least 24 weeks, and three or more sessions per week are characteristics associated with larger effects; these observations are hypothesis-generating rather than demonstrations of an optimal intervention model. For schools, the evidence supports structured physical activity as a promising strategy for selected fitness outcomes, whereas psychosocial benefits appear to depend more strongly on program design, theoretical grounding, and implementation quality. These cautiously positive findings support integrating comprehensive school-based PA programs into child health promotion strategies while underscoring the need for rigorous trials with standardized measurement to strengthen the evidence base.

## Data Availability

The original contributions presented in the study are included in the article/Supplementary material, further inquiries can be directed to the corresponding author.
