# Supplementary material for: Effects of school-based physical activity interventions on physical fitness and psychosocial adaptation in children: a systematic review and meta-analysis
Source: Front Public Health. 2026 Oct 2;14:1962774. doi: 10.3389/fpubh.2026.1962774 (PMC13672567; doi:10.3389/fpubh.2026.1962774)
Supplement: Supplementary file 1 [file Supplementary_file_1.docx]

**Supplementary Material S1**

**Complete Search Strategies for All Six Databases**

**Date of search: 15 January 2026**

No language or publication-type filters were applied. Hand-searching of reference lists of included studies and previous systematic reviews was also conducted.

**1. PubMed (via NLM)**

**Search string:**

("physical activity"[tiab] OR "exercise"[tiab] OR "physical fitness"[tiab] OR "physical education"[tiab] OR "school sport"[tiab]) AND ("school"[tiab] OR "school-based"[tiab] OR "school environment"[tiab] OR "primary school"[tiab] OR "elementary school"[tiab] OR "children"[tiab] OR "child"[tiab] OR "preadolescent"[tiab]) AND ("intervention"[tiab] OR "program"[tiab] OR "trial"[tiab] OR "randomized"[tiab] OR "controlled trial"[tiab] OR "RCT"[tiab]) AND ("cardiorespiratory fitness"[tiab] OR "muscular fitness"[tiab] OR "body composition"[tiab] OR "BMI"[tiab] OR "self-esteem"[tiab] OR "well-being"[tiab] OR "psychological"[tiab] OR "social"[tiab] OR "psychosocial"[tiab] OR "quality of life"[tiab])

**2. Web of Science Core Collection**

**Search string:**

TS=("physical activity" OR exercise OR "physical fitness" OR "physical education") AND TS=("school" OR "school-based" OR "primary school" OR "elementary school" OR children OR preadolescent) AND TS=(intervention OR program OR trial OR randomized OR "controlled trial") AND TS=("cardiorespiratory fitness" OR "muscular fitness" OR "body composition" OR BMI OR "self-esteem" OR well-being OR psychological OR social OR psychosocial OR "quality of life")

**3. Embase**

**Search string:**

'physical activity':ti,ab OR 'exercise':ti,ab OR 'physical fitness':ti,ab OR 'physical education':ti,ab AND 'school':ti,ab OR 'school-based':ti,ab OR 'primary school':ti,ab OR 'elementary school':ti,ab OR children:ti,ab OR preadolescent:ti,ab AND intervention:ti,ab OR program:ti,ab OR trial:ti,ab OR randomized:ti,ab OR 'controlled trial':ti,ab AND 'cardiorespiratory fitness':ti,ab OR 'muscular fitness':ti,ab OR 'body composition':ti,ab OR BMI:ti,ab OR 'self-esteem':ti,ab OR well-being:ti,ab OR psychological:ti,ab OR social:ti,ab OR psychosocial:ti,ab OR 'quality of life':ti,ab

**4. SPORTDiscus (EBSCOhost)**

**Search string:**

TX ("physical activity" OR exercise OR "physical fitness" OR "physical education") AND TX (school OR "school-based" OR "primary school" OR "elementary school" OR children OR preadolescent) AND TX (intervention OR program OR trial OR randomized OR "controlled trial") AND TX ("cardiorespiratory fitness" OR "muscular fitness" OR "body composition" OR BMI OR "self-esteem" OR well-being OR psychological OR social OR psychosocial OR "quality of life")

**5. Cochrane Central Register of Controlled Trials (CENTRAL)**

**Search string:**

("physical activity":ti,ab OR "exercise":ti,ab OR "physical fitness":ti,ab OR "physical education":ti,ab) AND ("school":ti,ab OR "school-based":ti,ab OR "primary school":ti,ab OR "elementary school":ti,ab OR "children":ti,ab OR "preadolescent":ti,ab) AND ("intervention":ti,ab OR "program":ti,ab OR "trial":ti,ab OR "randomized":ti,ab OR "controlled trial":ti,ab) AND ("cardiorespiratory fitness":ti,ab OR "muscular fitness":ti,ab OR "body composition":ti,ab OR "BMI":ti,ab OR "self-esteem":ti,ab OR "well-being":ti,ab OR "psychological":ti,ab OR "social":ti,ab OR "psychosocial":ti,ab OR "quality of life":ti,ab,kw)

**6. PsycINFO (APA)**

**Search string:**

("physical activity".ab. OR "exercise".ab. OR "physical fitness".ab. OR "physical education".ab.) AND ("school".ab. OR "school-based".ab. OR "primary school".ab. OR "elementary school".ab. OR "children".ab. OR "preadolescent".ab.) AND ("intervention".ab. OR "program".ab. OR "trial".ab. OR "randomized".ab. OR "controlled trial".ab.) AND ("cardiorespiratory fitness".ab. OR "muscular fitness".ab. OR "body composition".ab. OR "BMI".ab. OR "self-esteem".ab. OR "well-being".ab. OR "psychological".ab. OR "social".ab. OR "psychosocial".ab. OR "quality of life".ab.)

***Note.*** Searches were executed on 15 January 2026. No language or publication-type filters were applied at any stage. Records were exported to EndNote 21 (Clarivate), and duplicates were removed using both automated and manual screening. The complete PRISMA flow diagram (Figure 1) documents the number of records identified, screened, assessed for eligibility, and included from each source.
