# Supplementary material for: Effects of school-based physical activity interventions on physical fitness and psychosocial adaptation in children: a systematic review and meta-analysis
Source: Front Public Health. 2026 Oct 2;14:1962774. doi: 10.3389/fpubh.2026.1962774 (PMC13672567; doi:10.3389/fpubh.2026.1962774)
Supplement: Supplementary file 2 [file Supplementary_file_2.docx]

**Supplementary Material S2**

**Characteristics of Included Studies and Extracted Effect Sizes**

*This supplementary file provides the full characteristics of the 40 included studies (39 randomized controlled trials: 22 cluster-randomized and 17 individually randomized; 1 non-randomized controlled trial) and the study-level effect sizes extracted for each outcome domain. Table S2-1 reproduces the study-characteristics data summarized in Table 1 of the main manuscript; Table S2-2 reports the Hedges' g, standard error (SE), and 95% confidence interval (CI) for each study and outcome. Empty cells indicate that the study did not contribute data to that outcome domain.*

**Table S2-1. Characteristics of the 40 Included Studies**

| **Study (First Author, Year)** | **Country** | **Design** | **N** | **Age (years)** | **Intervention Type** | **Duration (weeks)** | **Frequency (sessions/week)** | **Physical Fitness Outcomes** | **Psychosocial Outcomes** | **RoB** |
| --- | --- | --- | --- | --- | --- | --- | --- | --- | --- | --- |
| Andermo et al., 2020 | Sweden | CRCT | 1,462 | 8–12 | Multicomponent (enhanced PE + active recess) | 24 | 3 | VO₂max, BMI | SDQ, RSES | Low |
| Ardoy et al., 2014 | Spain | RCT | 67 | 12–13 | Enhanced PE | 16 | 4 | 20-m SRT, BMI | — | Some concerns |
| Bailey et al., 2015 | UK | CRCT | 327 | 8–11 | Physically active learning | 12 | 3 | 20-m SRT | SDQ | Some concerns |
| Bartholomew et al., 2018 | USA | CRCT | 2,716 | 8–11 | Classroom PA breaks | 36 | 5 | — | SPPC, PANAS-C | Low |
| Burns et al., 2017 | Australia | RCT | 168 | 9–12 | Enhanced PE (FIT-2) | 20 | 3 | VO₂max, handgrip | — | Some concerns |
| Centeio et al., 2018 | USA | CRCT | 558 | 7–11 | Classroom PA breaks (CSPAP) | 12 | 5 | — | BESS | Some concerns |
| Chinapaw et al., 2015 | Netherlands | CRCT | 642 | 8–11 | Multicomponent (PE + classroom + environment) | 32 | 3 | 20-m SRT, BMI, WC | SPPC, SDQ | Low |
| Cohen et al., 2015 | Australia | CRCT | 460 | 8–10 | Enhanced PE (SCORES) | 12 | 3 | 20-m SRT, BMI | RSES | Some concerns |
| Costigan et al., 2016 | Australia | RCT | 65 | 13–14 | Enhanced PE (HIIT) | 8 | 3 | 20-m SRT, BMI | PANAS-C | Some concerns |
| Daly-Smith et al., 2018 | UK | CRCT | 596 | 9–11 | Physically active learning | 18 | 3 | — | SDQ, KIDSCREEN | Low |
| Donnelly et al., 2009 | USA | CRCT | 1,490 | 7–10 | Physically active learning (PAAC) | 104 | 5 | BMI | — | Low |
| Dudley et al., 2010 | Australia | CRCT | 193 | 12–13 | Enhanced PE | 20 | 3 | 20-m SRT, handgrip, BMI | SPPC | Some concerns |
| Eather et al., 2016 | Australia | RCT | 96 | 12–13 | Enhanced PE (CrossFit Teens) | 8 | 2 | 20-m SRT, BMI, WC | RSES | Some concerns |
| Erwin et al., 2012 | USA | CRCT | 229 | 8–11 | Classroom PA breaks | 20 | 5 | — | BESS | Some concerns |
| Fairclough et al., 2016 | UK | CT | 328 | 10–11 | Active recess | 12 | 3 | 20-m SRT, BMI | SDQ | Some concerns |
| Fu et al., 2021 | China | CRCT | 637 | 8–11 | Multicomponent (PE + classroom + recess) | 16 | 4 | 20-m SRT, BMI, handgrip | — | Low |
| Goh et al., 2019 | USA | RCT | 108 | 9–12 | Classroom PA breaks | 12 | 5 | — | PANAS-C | Some concerns |
| Graf et al., 2008 | Germany | RCT | 668 | 7–9 | Enhanced PE + health education | 48 | 3 | 20-m SRT, BMI, WC | — | Low |
| Grieco et al., 2016 | USA | RCT | 320 | 8–10 | Physically active learning | 16 | 3 | BMI | — | Some concerns |
| Haapala et al., 2014 | Finland | RCT | 82 | 7–9 | Enhanced PE + active recess | 24 | 5 | 20-m SRT, BMI | SPPC | Some concerns |
| Hartmann et al., 2010 | Switzerland | RCT | 258 | 8–12 | Enhanced PE | 32 | 5 | 20-m SRT, handgrip | — | Low |
| Howie et al., 2014 | USA | RCT | 96 | 9–12 | Classroom PA breaks | 12 | 5 | — | PANAS-C | Some concerns |
| Kallio et al., 2016 | Finland | CRCT | 352 | 9–12 | Enhanced PE | 24 | 3 | — | SDQ, KIDSCREEN | Some concerns |
| Kriemler et al., 2010 | Switzerland | CRCT | 540 | 7–8 | Multicomponent (PE + breaks + family) | 24 | 5 | VO₂max, 20-m SRT, BMI | SDQ | Low |
| Lai et al., 2014 | China | CRCT | 921 | 8–12 | Enhanced PE (Happy 10) | 32 | 5 | 20-m SRT, BMI, WC | — | Some concerns |
| Lonsdale et al., 2013 | Australia | CRCT | 527 | 12–14 | Enhanced PE (SDT-based) | 12 | 3 | 20-m SRT | — | Low |
| Magnusson et al., 2011 | Iceland | RCT | 326 | 7–9 | Enhanced PE + health education | 24 | 3 | 20-m SRT, BMI | SPPC | Some concerns |
| Martinez-Vizcaino et al., 2014 | Spain | CRCT | 1,192 | 8–11 | Multicomponent (PE + active breaks) | 32 | 4 | 20-m SRT, BMI, WC | — | Low |
| Mayorga-Vega et al., 2017 | Spain | RCT | 154 | 10–12 | Enhanced PE | 8 | 2 | 20-m SRT | — | Some concerns |
| Mullender-Wijnsma et al., 2016 | Netherlands | CRCT | 499 | 8–10 | Physically active learning | 24 | 3 | 20-m SRT, BMI | SDQ | Low |
| Norris et al., 2018 | UK | CRCT | 347 | 9–11 | Enhanced PE | 12 | 3 | — | SPPC, SDQ | Some concerns |
| Resaland et al., 2011 | Norway | CRCT | 256 | 10–11 | Enhanced PE | 24 | 5 | VO₂max, 20-m SRT, BMI, WC | — | Low |
| Sacchetti et al., 2013 | Italy | RCT | 215 | 8–10 | Enhanced PE | 24 | 2 | 20-m SRT, handgrip, BMI | RSES | Some concerns |
| Sallis et al., 1997 | USA | CRCT | 955 | 9–10 | Enhanced PE (SPARK) | 24 | 3 | 20-m SRT, BMI | — | Low |
| Salmon et al., 2008 | Australia | RCT | 311 | 10–12 | Enhanced PE + classroom | 20 | 3 | BMI | SPPC | Some concerns |
| Sanchez-Lopez et al., 2019 | Spain | CRCT | 1,478 | 7–10 | Multicomponent (MOVI-KIDS) | 24 | 3 | 20-m SRT, BMI, WC, handgrip | SDQ | Low |
| Thivel et al., 2011 | France | RCT | 457 | 7–10 | Enhanced PE + active recess | 24 | 4 | 20-m SRT, BMI, WC | — | Some concerns |
| Toftager et al., 2014 | Denmark | CRCT | 1,341 | 12–13 | Multicomponent (PE + environment) | 26 | 4 | 20-m SRT, BMI | — | Low |
| Vazou et al., 2016 | USA | RCT | 168 | 8–11 | Classroom PA breaks | 8 | 5 | — | PANAS-C, BESS | Some concerns |
| Walther et al., 2014 | Germany | RCT | 188 | 11–12 | Enhanced PE (daily PE) | 48 | 5 | VO₂max, 20-m SRT, BMI | — | Low |

**Table S2-2. Study-Level Effect Sizes (Hedges' g) by Outcome Domain**

| **Study** | **CRF: g (SE) [95% CI]** | **Muscular: g (SE) [95% CI]** | **BMI: g (SE) [95% CI]** | **Self-esteem: g (SE) [95% CI]** | **Well-being: g (SE) [95% CI]** | **Social: g (SE) [95% CI]** |
| --- | --- | --- | --- | --- | --- | --- |
| Andermo et al., 2020 | -0.179 (0.053) [-0.283, -0.076] | — | 0.768 (0.052) [0.665, 0.87] | -0.026 (0.052) [-0.129, 0.076] | -0.045 (0.052) [-0.148, 0.058] | 0.171 (0.052) [0.068, 0.274] |
| Ardoy et al., 2014 | -0.479 (0.247) [-0.962, 0.005] | 0.969 (0.246) [0.487, 1.45] | 0.982 (0.245) [0.503, 1.461] | — | 0.185 (0.245) [-0.296, 0.666] | — |
| Bailey et al., 2015 | — | 0.51 (0.111) [0.292, 0.727] | 1.418 (0.111) [1.201, 1.635] | -0.352 (0.111) [-0.569, -0.135] | — | — |
| Bartholomew et al., 2018 | 0.886 (0.039) [0.81, 0.962] | — | — | — | — | — |
| Burns et al., 2017 | -0.155 (0.156) [-0.46, 0.15] | 0.455 (0.155) [0.151, 0.759] | -0.904 (0.154) [-1.207, -0.602] | 0.58 (0.155) [0.277, 0.883] | — | — |
| Centeio et al., 2018 | 0.821 (0.085) [0.653, 0.988] | — | 0.48 (0.085) [0.313, 0.646] | 0.411 (0.085) [0.244, 0.577] | — | — |
| Chinapaw et al., 2015 | 0.581 (0.08) [0.425, 0.737] | — | — | — | -0.188 (0.079) [-0.343, -0.032] | — |
| Cohen et al., 2015 | 0.587 (0.094) [0.403, 0.772] | 0.43 (0.094) [0.246, 0.614] | -0.462 (0.093) [-0.645, -0.279] | — | 0.788 (0.094) [0.605, 0.972] | — |
| Costigan et al., 2016 | -0.439 (0.25) [-0.93, 0.051] | 0.503 (0.249) [0.014, 0.992] | 0.521 (0.248) [0.034, 1.007] | — | 1.161 (0.249) [0.673, 1.649] | — |
| Daly-Smith et al., 2018 | — | 0.059 (0.082) [-0.103, 0.22] | — | -0.179 (0.082) [-0.34, -0.019] | — | — |
| Donnelly et al., 2009 | 0.574 (0.052) [0.471, 0.676] | — | -0.598 (0.052) [-0.7, -0.497] | — | — | — |
| Dudley et al., 2010 | -0.076 (0.145) [-0.361, 0.209] | — | — | 0.691 (0.144) [0.408, 0.974] | — | -0.493 (0.144) [-0.776, -0.21] |
| Eather et al., 2016 | -0.281 (0.206) [-0.685, 0.122] | -0.258 (0.205) [-0.66, 0.144] | — | — | -0.819 (0.205) [-1.22, -0.417] | — |
| Erwin et al., 2012 | 0.815 (0.133) [0.554, 1.077] | — | -0.486 (0.132) [-0.745, -0.227] | — | 0.922 (0.133) [0.662, 1.182] | — |
| Fairclough et al., 2016 | -0.28 (0.111) [-0.498, -0.061] | 0.193 (0.111) [-0.025, 0.41] | — | — | — | 0.229 (0.111) [0.012, 0.447] |
| Fu et al., 2021 | 1.184 (0.08) [1.028, 1.341] | — | -1.12 (0.079) [-1.275, -0.965] | 0.173 (0.079) [0.017, 0.329] | 0.001 (0.08) [-0.155, 0.157] | — |
| Goh et al., 2019 | 0.179 (0.194) [-0.202, 0.559] | — | -0.464 (0.193) [-0.841, -0.086] | 0.466 (0.193) [0.088, 0.844] | — | -0.354 (0.193) [-0.732, 0.024] |
| Graf et al., 2008 | — | -0.249 (0.078) [-0.401, -0.096] | -0.016 (0.077) [-0.168, 0.135] | — | — | — |
| Grieco et al., 2016 | 0.264 (0.113) [0.043, 0.486] | — | -0.603 (0.112) [-0.822, -0.383] | -0.272 (0.112) [-0.492, -0.052] | — | — |
| Haapala et al., 2014 | — | — | — | -0.074 (0.221) [-0.508, 0.36] | -0.256 (0.222) [-0.691, 0.178] | — |
| Hartmann et al., 2010 | -0.089 (0.126) [-0.335, 0.157] | 0.371 (0.125) [0.126, 0.616] | -0.244 (0.125) [-0.488, 0.0] | — | -0.049 (0.125) [-0.294, 0.195] | 0.541 (0.125) [0.296, 0.786] |
| Howie et al., 2014 | 0.51 (0.206) [0.106, 0.913] | 0.965 (0.205) [0.563, 1.367] | -0.085 (0.204) [-0.485, 0.316] | -0.196 (0.205) [-0.596, 0.205] | — | — |
| Kallio et al., 2016 | — | — | 0.68 (0.107) [0.471, 0.889] | 0.256 (0.107) [0.047, 0.465] | — | 0.262 (0.107) [0.053, 0.472] |
| Kriemler et al., 2010 | 0.299 (0.087) [0.128, 0.469] | 0.088 (0.087) [-0.081, 0.258] | — | — | — | — |
| Lai et al., 2014 | 0.042 (0.066) [-0.088, 0.172] | 0.465 (0.066) [0.336, 0.595] | -1.109 (0.066) [-1.239, -0.98] | — | — | -0.429 (0.066) [-0.558, -0.299] |
| Lonsdale et al., 2013 | 1.044 (0.088) [0.872, 1.216] | -0.127 (0.088) [-0.299, 0.044] | -0.347 (0.087) [-0.518, -0.176] | — | — | — |
| Magnusson et al., 2011 | 1.587 (0.112) [1.368, 1.806] | — | -0.068 (0.111) [-0.286, 0.149] | 0.279 (0.111) [0.061, 0.496] | — | -0.054 (0.111) [-0.272, 0.163] |
| Martinez-Vizcaino et al., 2014 | 0.349 (0.058) [0.234, 0.464] | — | — | 0.477 (0.058) [0.363, 0.591] | — | — |
| Mayorga-Vega et al., 2017 | — | 0.019 (0.162) [-0.299, 0.336] | 0.442 (0.161) [0.126, 0.758] | — | — | — |
| Mullender-Wijnsma et al., 2016 | -0.135 (0.09) [-0.312, 0.042] | 0.286 (0.09) [0.109, 0.462] | 0.414 (0.09) [0.238, 0.59] | 0.003 (0.09) [-0.173, 0.179] | 0.611 (0.09) [0.435, 0.787] | 0.013 (0.09) [-0.163, 0.189] |
| Norris et al., 2018 | 0.311 (0.108) [0.099, 0.523] | — | — | — | — | 0.283 (0.108) [0.072, 0.494] |
| Resaland et al., 2011 | — | 0.917 (0.126) [0.67, 1.163] | -0.523 (0.125) [-0.768, -0.278] | 0.429 (0.125) [0.184, 0.675] | — | — |
| Sacchetti et al., 2013 | 0.915 (0.138) [0.645, 1.185] | 0.836 (0.137) [0.567, 1.105] | 0.389 (0.137) [0.122, 0.657] | — | — | — |
| Sallis et al., 1997 | 1.128 (0.065) [1.0, 1.256] | — | -0.255 (0.065) [-0.382, -0.128] | — | -0.171 (0.065) [-0.298, -0.044] | 0.511 (0.065) [0.384, 0.638] |
| Salmon et al., 2008 | -0.177 (0.114) [-0.402, 0.047] | 0.374 (0.114) [0.151, 0.598] | -1.178 (0.114) [-1.401, -0.956] | 0.218 (0.114) [-0.004, 0.441] | 0.023 (0.114) [-0.2, 0.246] | — |
| Sanchez-Lopez et al., 2019 | — | — | -0.126 (0.052) [-0.228, -0.024] | — | — | — |
| Thivel et al., 2011 | -0.016 (0.094) [-0.201, 0.169] | -0.321 (0.094) [-0.506, -0.137] | — | — | — | — |
| Toftager et al., 2014 | 0.275 (0.055) [0.167, 0.383] | — | -0.188 (0.055) [-0.296, -0.081] | 0.408 (0.055) [0.3, 0.515] | 0.343 (0.055) [0.236, 0.451] | 1.043 (0.055) [0.935, 1.15] |
| Vazou et al., 2016 | 0.267 (0.156) [-0.038, 0.573] | -0.278 (0.155) [-0.582, 0.026] | — | — | — | 0.82 (0.155) [0.516, 1.123] |
| Walther et al., 2014 | 0.107 (0.147) [-0.181, 0.395] | 0.09 (0.147) [-0.197, 0.377] | — | — | — | — |

*Abbreviations: CRF, cardiorespiratory fitness; BMI, body mass index; CI, confidence interval; g, Hedges' g; SE, standard error. Negative g values favour the control condition for BMI (i.e., lower BMI in the intervention group) and favour the intervention for positively framed outcomes as defined in the main manuscript.*
